# Improved biolistic transformation and genome editing in wheat by using trehalose for high osmotic treatment

**DOI:** 10.5511/plantbiotechnology.24.0328a

**Published:** 2024-06-25

**Authors:** Chizu Yanagihara, Hiroshi Tsukamoto, Yuji Ishida, Toshihiko Komari

**Affiliations:** 1 Plant Innovation Center, Japan Tobacco Inc.; 2 Agri-Bio Research Center, KANEKA CORPORATION

**Keywords:** genome editing, particle bombardment, transformation, trehalose, wheat

## Abstract

The tissue culture process is usually involved in gene transfer and genome editing in plants. Like other species, there is enormous variation among wheat genotypes in tissue culture response. In the rapidly advancing system of CRISPR/Cas9 for genome editing, particle bombardment has received increasing attention as a delivery method for a large amount of nucleic acids and RNA-protein complexes. However, the efficiency of transformation by particle bombardment has been low in wheat, and only a limited number of varieties have been transformed. In this study, replacement of maltose with trehalose as an osmolyte for high osmotic treatment for the protection of tissues from physical impacts improved callus formation in immature wheat embryos and efficiency of transformation and genome editing in varieties that are relatively poor in tissue culture response. The range of varieties amenable to biolistic transformation and genome editing may be expanded by this modification.

Tissue culture is a base technology for gene transfer and genome editing, but in every plant species, there is considerable variation among genotypes in amenability in tissue culture and plant regeneration from cultured cells. Various factors have been studied to overcome the technical hurdles. For example, wheat transformation efficiency was improved by the modification of media, such as the addition of CuSO_4_ and AgNO_3_ to the medium for co-cultivation with *Agrobacterium tumefaciens*; changes to the gelling agent; and the addition of handling steps, such as centrifugation of immature embryos before inoculation and the excision of embryo axes after inoculation (Ishida et al. 2015). Although *Agrobacterium*-mediated transformation has been more popular because a small number of copies of relatively large DNA fragments with defined ends can be transferred to plant chromosomes with few rearrangements (Wang et al. 2018), particle bombardment has received increasing attention as a method of delivery of a large amount of DNA, RNA, and RNA-protein complexes for the rapidly advancing system of CRISPR/Cas9 for genome editing. However, the efficiency of transformation by particle bombardment has been low in wheat, and only a limited number of varieties have been transformed. Bombarded tissues are usually placed on high-osmotic media to be protected from physical impacts. Often, more than twice as much maltose as in typical culture media is added to the media. In this study, trehalose, which is involved in stress response in plants (Fernandez et al. 2010), was compared with maltose. Replacement of maltose with trehalose elevated the efficiency of transformation and genome editing, especially in wheat varieties that are relatively poor in tissue culture response.

Seeds of the wheat variety Fielder were obtained from Kihara Institute for Biological Research, Yokohama City University (Accession number KT020-061). Seeds of the varieties Mace, Cadenza, Paragon, Claire, and Jagger were obtained from a commercial distributer. These varieties are listed in “The 10+ Wheat Genomes Project” (https://www.seedstor.ac.uk/index.php (May 31st, 2024)) and are also available from “The Germplasm Resources Unit” (https://www.seedstor.ac.uk/index.php (May 31st, 2024)). These varieties were grown at 26°C during the day and 17°C at night in a greenhouse. Day length was controlled for 16 h with supplemental light (metal halide lamps). Immature embryos were isolated between 14 and 16 days after anthesis and centrifuged at 5,300×g according to Ishida et al. (2015). In the subsequent steps, the media described by Ishida et al. (2015) or media partially modified from those described were used. The embryo axes were excised. For maltose treatment, 25 of the embryos were placed on 9-cm plates of high-osmotic WLS90-Res medium, which was WLS-Res medium modified by increasing the maltose monohydrate concentration to 90 g l^−1^ (250 mM) according to Rasco-Gaunt et al. (2001). For trehalose treatment, 25 embryos were placed on 9-cm plates of WLS0/90-Res medium, which was WLS90-Res medium modified by replacing maltose monohydrate with 94.6 g l^−1^ (250 mM) of trehalose dihydrate. In both treatments, the embryos were incubated for pre-culture at 26°C in the dark for one day.

As a preliminary step, maltose and trehalose treatments were compared in culture experiments involving two varieties, Mace and Paragon, without the bombardment process. The pre-culture was prolonged for four more days. The embryos were incubated on WLS90-Res medium for resting culture at 25°C in the dark for 10 days; on WLS60 medium, which was WLS medium modified by increasing the maltose monohydrate concentration to 60 g l^−1^, for the first callus culture at 25°C in the dark for 10 days; and on WLS medium for the second callus culture at 25°C in the dark for 14 days. Regeneration and rooting cultures followed the method of Ishida et al. (2015) with the following modifications: LSZ-P5 and LSF-P5 media were replaced with LSZ and LSF, respectively. As shown in Table 1, the frequencies of callus formation and plant regeneration from the trehalose treatment were significantly higher than those from the maltose treatment.

**Table 1:** Table 1. Callus formation and plant regeneration without particle bombardment.

Variety	Sugar for high osmotic treatment	Number of embryos tested (a)	Embryos from which calli were formed			Embryos from which plants were regenerated		
			Number (b)	b/a (%)	p*	Number (c)	c/a (%)	p*
Mace	Maltose	100	57	57.0	0.002	49	49.0	0.004
	Trehalose	50	41	82.0		37	74.0	
Paragon	Maltose	100	49	49.0	<0.001	42	42.0	<0.001
	Trehalose	50	40	80.0		40	80.0	

* Maltose and trehalose treatments were compared by Chi-square test.

Subsequently, maltose and trehalose treatments were compared in gene transfer and genome editing by particle bombardment. Expression cassettes for the Cas9, bar, and guide RNA genes were constructed as shown in Figure 1. The Cas9 gene was chemically synthesized according to the sequence modified by Svitashev et al. (2015) to optimize the codon usage and to add an intron and connected to the maize ubiquitin promoter including the first intron and to the nos and 35S terminators. The bar gene, which encodes for a phosphinothricin acetyl transferase gene, was connected to the 35S promoter and to the nos terminator. The Cas9 and bar cassettes were inserted into pUC19 to yield pUbi-Cas9 and p35ScatI-bar, respectively. The guide RNA targeted to the *TaLOX2* gene (Zhang et al. 2016) was inserted between the TaU6 promoter and terminator on pUC18 to yield pTaU6-LOX2-S1-gRNA. For a shot of bombardment described below, 100 fmol of p35ScatI-bar, 30 fmol of pUbi-Cas9, and 30 fmol of pTaU6-LOX2-S1-gRNA were mixed in 3.3 µl of plasmid DNA solution.

**Figure 1:**
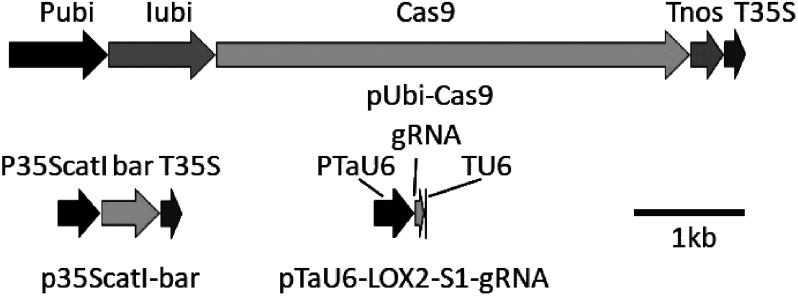
Figure 1. Maps of expression cassettes. Cas9, bar, and gRNA expression cassettes were cloned into pUC19, pUC19, and pUC18, respectively. Abbreviations: Pubi, maize ubiquitin gene promoter; Iubi, the first intron of the maize ubiquitin gene; Tnos, nopaline synthase terminator; P35S, Cauliflower Mosaic Virus (CaMV) 35S promoter; catI, catalase intron; bar, Basta resistance; T35S, CaMV 35S terminator; PTaU6, TaU6 promoter; gRNA, single-guide RNA targeting the *TaLOX2* gene; TU6, TaU6 terminator.

The embryos of six wheat varieties—Fielder, Mace, Cadenza, Paragon, Claire, and Jagger—on the pre-culture plates were bombarded by PDS-1000 (BIO-RAD) according to the protocol described by Ishida et al. (2020) except for the following modifications: the gold particles were suspended at density of 40 mg ml^−1^, and 3.3 µl of the suspension was mixed with the 3.3 µl of plasmid DNA solution and 0.12 µl of TransiT-2020 (TaKaRa) by vortex for 2–3 min. The particles in the mixture were pelleted by centrifugation, washed with 70% ethanol and 100% ethanol, suspended in 5 µl of absolute ethanol, sonicated for 30 s, and applied to a macro carrier for a shot of bombardment. The bombarded plates were incubated at 25°C in the dark for one day and at 28°C in the dark for 3 days. Then, the resting, the first callus, and the second callus cultures described above were performed with the following modifications: WLS60 and WLS were replaced with WLS60-P5 medium, which was WLS-P5 medium modified by increasing maltose monohydrate to 60 g l^−1^, and WLS-P10, respectively. Regeneration and rooting followed the method of Ishida et al. (2015).

As shown in Table 2, the frequencies of both callus formation and plant regeneration from the trehalose treatment were significantly higher than those from the maltose treatment in four varieties: Mace, Paragon, Claire, and Cadenza. The difference between the trehalose and maltose treatments was small in Fielder, which is the best variety in tissue culture response in wheat (Ishida, personal communication). Very few calli and no regenerants were obtained from the trehalose or maltose treatment in Jagger, which is the poorest among the six varieties in tissue culture response (Ishida, personal).

**Table 2:** Table 2. Callus formation, plant regeneration, and mutants from bombarded embryos.

Variety	Sugar for high osmotic treatment	Number of embryos tested (a)	Embryos from which calli were formed			Embryos from which plants were regenerated			Embryos from which mutants were produced			Mutants per regenerant	
			Number (b)	b/a (%)	p*	Number (c)	c/a (%)	p*	Number (d)	d/a (%)	p*	d/c (%)	p*
Fielder	Maltose	425	184	43.3	0.059	73	17.2	0.220	29	6.8	0.046	39.7	0.113
	Trehalose	425	157	36.9		60	14.1		16	3.8		26.7	
Mace	Maltose	500	171	34.2	<0.001	23	4.6	<0.001	6	1.2	<0.001	26.1	0.198
	Trehalose	500	286	57.2		119	23.8		48	9.6		40.3	
Paragon	Maltose	525	54	10.3	<0.001	6	1.1	<0.001	1	0.2	<0.001	16.7	0.555
	Trehalose	525	191	36.4		61	11.6		17	3.2		27.9	
Claire	Maltose	250	51	20.4	<0.001	10	4.0	<0.001	3	1.2	0.007	30.0	0.673
	Trehalose	75	35	46.7		13	17.3		5	6.7		38.5	
Cadenza	Maltose	200	81	40.5	<0.001	2	1.0	0.024	1	0.5	0.616	50.0	0.171
	Trehalose	50	32	64.0		3	6.0		0	0.0		0.0	
Jagger	Maltose	250	1	0.4	0.365	0	0.0	>0.999	0	0.0	>0.999	—	—
	Trehalose	75	1	1.3		0	0.0		0	0.0		—	

* Maltose and trehalose treatments were compared by Chi-square test.

It is likely that trehalose in the high-osmotic medium elevated transformation and editing efficiency by improving tissue culture response of the embryos in some of the varieties. As trehalose did not increase the transformation frequency of Fielder, which is the least recalcitrant variety tested in this study, trehalose may be particularly effective in varieties that are relatively poor in culture response. It might be possible that the scale of the experiment was not enough to study the effects on Jagger, the most recalcitrant variety tested in this study.

Mutations in the *TaLOX2* gene of the A, B, and D sub-genomes were detected by CAPS analysis described by Zhang et al. (2016) of DNA extracted using QIAcube HT (QIAGEN) from 5–10 mg of frozen leaves ground in a Tissue LyserII (QIAGEN). As shown in Table 2, the frequencies of mutants per embryo from the trehalose treatment were significantly higher than those from the maltose treatment in three varieties Mace, Paragon, and Claire. The frequency of mutants from the trehalose treatment was lower than that from the maltose treatment in Fielder, and only one mutant was obtained in Cadenza. On the other hand, the frequencies of mutants per regenerant did not differ significantly between the treatments in any of the varieties partially because the number of the regenerants were small in many of the plots.

Some of the mutant plants were examined further by PCR amplification using the primers listed in Table 3 for the presence of the cassettes (Table 4). Bar was detected in almost all of the tested plants, confirming that they were mostly transgenic plants. In some of the tested plants, neither the Cas9 gene nor the guide RNA sequence was detected, suggesting that genome editing was caused by transient expression of these genes. Some of the mutations in Claire and Mace were also sequenced using SeqStudio Genetic Analyzer from Applied Biosystems (Table 5). Like many of the mutations caused by Cas9 in wheat previously reported (Zhang et al. 2016), short insertions, short deletions, or combinations of them were found at locations close to the protospacer-adjacent motif (PAM) sequences in one or more alleles. There were no apparent differences in the genome editing patterns between the plants from the maltose and trehalose treatments.

**Table 3:** Table 3. Primer list.

Target	Primer name	Sequence	Product
Cas9	Cas9 Tool free REC1 16	5′-CACCGCCTGGAGGAATCATTCCTG-3′	816 bp
	Cas9 Tool free REC2 1	5′-CCGAAGGATGTCGCTGAGCAGGATAG-3′	
Cas9	Cas9 Tool free RuvCII 1	5′-GACTCCCTCCACGAACACATCGCC-3′	1094 bp
	Cas9 Tool free RuvCIII 50	5′-GTCGCGAAATCCCGGCCCTTATC-3′	
Cas9	Cas9 Tool free PI 201	5′-GATCACGATTATGGAGCGGTCCTCCTTC-3′	526 bp
	Cas9 Tool free PI Rv142	5′-CTTCGTGCTCGTGTACCGCTTCCG-3′	
Guide RNA	gRNA Tool free pTaU6 1	5′-GACCAAGCCCGTTATTCTGAC-3′	434 bp
	gRNA Tool free ter 31–61	5′-CAAGTTGATAACGGACTAGCCTTATTTTAAC-3′	
Bar	Bar 89–107F	5′-GAGACAAGCACGGTCAACTT-3′	248 bp
	Bar 317–335R	5′-TTCAGCAGGTGGGTGTAGA-3′	

**Table 4:** Table 4. Detection of integrated transgenes by PCR.

Variety	Sugar for high osmotic treatment	Number of plants analyzed (a)	Cas9 detected			Guide RNA gene detected			Bar detected		
			Number (b)	b/a (%)	p*	Number (c)	c/a (%)	p*	Number (d)	d/a (%)	p*
Fielder	Maltose	17	5	29.4	0.022	11	64.7	0.09	16	94.1	0.543
	Trehalose	6	5	83.3		6	100.0		6	100.0	
Mace	Maltose	4	1	25.0	0.162	3	75.0	0.86	4	100.0	>0.999
	Trehalose	19	12	63.2		15	78.9		19	100.0	
Claire	Maltose	3	0	0.0	0.028	1	33.3	0.04	3	100.0	>0.999
	Trehalose	5	4	80.0		5	100.0		5	100.0	

* Maltose and trehalose treatments were compared by Chi-square test.

**Table 5:** Table 5. Sequence of the regions around the target gene in 12 mutants.

Variety	Plant No.	Osmolyte in pre- and post-bombardment culture	Genome	Sequence	In/Del
Claire	Wild type	—	A, B, D	TACGTGCCGCGCGACGAGCT-------CTTCGGCCACCTCAAG	—
	Cl1	Maltose	D	TACGTGCCGCGCGACGAG··-------··············AG	−16
	Cl2	Maltose	B	TACGTGCCGCGCGACGAG··-------CTTCGGCCACCTCAAG	−2
	Cl2	Maltose	B	TACGTGCCGCGCGAC·····-----ttCTTCGGCCACCTCAAG	−5, +2
	Cl4	Maltose	D	TACGTGCCGCGCGACGAGC·----attCTTCGGCCACCTCAAG	−1, +3
	Cl5	Trehalose	B	TACGTGCCGCGCGACGAG··-------······aCACCTCAAG	−9, +1
	Cl7	Trehalose	A	TACGTGCCGCGCGACGAGCT------aCTTCGGCCACCTCAAG	+1
	Cl8	Trehalose	B	TACGTGCCGCGCGACGAG··-------CTTCGGCCACCTCAAG	−2
	Cl8	Trehalose	D	TACGTGCCGCGCGA······-------CTTCGGCCACCTCAAG	−6
	Cl8	Trehalose	D	TACGTGCCGCGCGACGAGC·-------CTTCGGCCACCTCAAG	−1
Mace	Wild type	—	A, B, D	TACGTGCCGCGCGACGAGCT-------CTTCGGCCACCTCAAG	—
	Ma1	Maltose	B	TACGTGCCGCGCGACGAGCT------aCTTCGGCCACCTCAAG	+1
	Ma6	Maltose	B	TACGTGCCGCGCGA······-------CTTCGGCCACCTCAAG	−6
	Ma7	Maltose	A	TACGTGCCGCGCGACG····-------CTTCGGCCACCTCAAG	−4
	Ma7	Maltose	D	TACGTGCCGCGCGACGAGCT------aCTTCGGCCACCTCAAG	+1
	Ma7	Maltose	D	TACGTGCCGC··········------tggtgGGCCACCTCAAG	−14, +5
	Ma8	Maltose	A	TACGTGCCGCGCGACGAG··-------CTTCGGCCACCTCAAG	−2
	Ma8	Maltose	B	TACGTGCCGCGCGACGAG··-------CTTCGGCCACCTCAAG	−2
	Ma2	Trehalose	D	TACGTGCCGCGCGACGAGCT------cCTTCGGCCACCTCAAG	+1
	Ma2	Trehalose	D	TACGTGCCGCGCGACGAG··−65 bp-CTTCGGCCACCTCAAG	−2, +65
	Ma3	Trehalose	A	TACGTGCCGCGCGACGAG··-------CTTCGGCCACCTCAAG	−2
	Ma3	Trehalose	B	TACGTGCCGCGCGACGAG··-------CTTCGGCCACCTCAAG	−2

The protospacer-adjacent motif (PAM) sequence is indicated by underline. Insertions are indicated by small letters except for the 65 bp insertion in Ma2, which was from pUC plasmid (1657–1721 of GenBank Accession L08752.1). Deletions are indicated by middle dots.

While trehalose is known to inhibit plant metabolism and growth (Frison et al. 2007; Müller et al. 2001), positive effects in tissue culture were reported in some species. In *Cymbidium finlaysonianum*, development of protocorm-like bodies was promoted by the addition of trehalose to the culture medium (Shimasaki et al. 2003). In torenia, trehalose extended the culture period for the maintenance of plant materials that required vegetative propagation without a reduction in plant viability (Yamaguchi et al. 2011). However, trehalose did not elevate plant regeneration from cultured microspores in wheat (Redha and Suleman 2013). Promotion by trehalose of plant callus formation or regeneration from cultured cells has not been reported to date. This study suggests a novel application of trehalose, which, in the high osmotic culture, may significantly improve biolistic transformation and genome editing protocols in various wheat genotypes and other crops.

## Abbreviations

bar: Basta resistance
CAPS: cleaved amplified polymorphic sequence
Cas9: CRISPR-associated protein 9
catI: catalase intron
CRISPR: clustered regularly interspaced short palindromic repeats
LOX: lipoxygenase
nos: nopaline synthase
ubi: ubiquitin
